# Emerging roles of MITF as a crucial regulator of immunity

**DOI:** 10.1038/s12276-024-01175-5

**Published:** 2024-02-13

**Authors:** Aram Lee, Jihyun Lim, Jong-Seok Lim

**Affiliations:** Department of Biological Science and the Cellular Heterogeneity Research Center, Research Institute of Women’s Health, Sookmyung Women’s University, Seoul, 04310 Republic of Korea

**Keywords:** Tumour immunology, Drug development

## Abstract

Microphthalmia-associated transcription factor (MITF), a basic helix-loop-helix leucine zipper transcription factor (bHLH-Zip), has been identified as a melanocyte-specific transcription factor and plays a critical role in melanocyte survival, differentiation, function, proliferation and pigmentation. Although numerous studies have explained the roles of MITF in melanocytes and in melanoma development, the function of MITF in the hematopoietic or immune system—beyond its function in melanin-producing cells—is not yet fully understood. However, there is convincing and increasing evidence suggesting that MITF may play multiple important roles in immune-related cells. Therefore, this review is focused on recent advances in elucidating novel functions of MITF in cancer progression and immune responses to cancer. In particular, we highlight the role of MITF as a central modulator in the regulation of immune responses, as elucidated in recent studies.

## MITF: The Unexpected Regulator of Immune Responses in Cancer

Understanding the role of a gene named MITF in diseases and immune responses is vital, as its functions are not fully understood. MITF is known to affect our skin color and the development of melanoma. However, its involvement in other diseases and its impact on our immune system are still under investigation. This study investigates how MITF interacts with immune cells, and how it might influence treatments for diseases like melanoma. The key findings reveal that MITF has a complex relationship with the immune system. The researchers propose that understanding MITF’s role in the immune system could lead to improved treatments for cancer and other diseases. They believe that targeting MITF might enhance the efficacy of current therapies and assist in developing new ones. The potential future implications of this research could be significant for improving patient outcomes in various diseases.

This summary was initially drafted using artificial intelligence, then revised and fact-checked by the author.

## Introduction

**Fig. 1:**
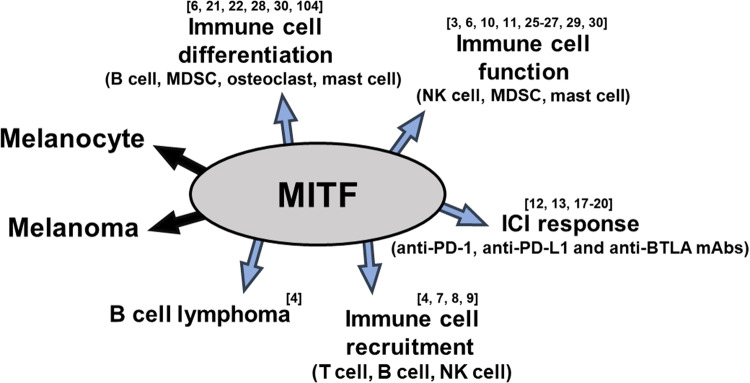
The schematic shows the multiple roles of MITF. MITF has been implicated in numerous immunological events in immune-related diseases, including cancer; the ICI response; and the differentiation, function and recruitment of immune cells (e.g., T cells, B cells, NK cells, MDSCs, osteoclasts and mast cells).

Since the discovery of the role of MITF (microphthalmia-associated transcription factor) in melanogenesis, the function of MITF has been reported in the context of several diseases, mostly in melanin-related diseases and melanoma^1–3^. However, the correlations of MITF expression with immune responses in patients with immunological diseases or cancers, including melanoma, and with the response to immune checkpoint inhibitors (ICIs) used to treat various tumor types have recently been investigated (Fig. 1).

### Association of MITF expression with the progression of cancer and other diseases

There is strong evidence indicating that MITF is involved in several diseases, including cancer, although the precise mechanisms and roles of MITF in these diseases remain largely unclear. Indeed, MITF expression has been observed in patients with diffuse large B-cell lymphoma (DLBCL), and it has been reported that high MITF expression in macrophages is associated with poor overall survival^4^. Copper plays a critical role in several biological processes involved in normal growth, development and immune responses and is associated with several diseases, including cancer, Alzheimer’s disease, MEDNIK syndrome and Menkes disease^5^. Mast cells (MCs) contain numerous highly electron-dense secretory granules, and the abundance of these granules is increased in several pathological conditions. The release of β-tryptase from these secretory granules is a hallmark of mast cell degranulation. Interestingly, copper inversely regulates the expression of MITF and its downstream target, the tryptase gene (Mcpt6), by inducing ERK1/2 phosphorylation. Notably, an increase in the number of tryptase-positive mast cells (MCs) in the skin and low copper levels in serum have been observed in patients with MEDNIK syndrome^6^.

In melanoma, MITF expression affects the recruitment of immune cells, including T cells, B cells, natural killer (NK) cells and myeloid cells, by altering the levels of chemokines and cytokines and ultimately altering therapeutic efficacy^7,8^. Similarly, increased infiltration of T cells, cytotoxic T cells (CTLs), NK cells and B cells has been found in MITF^high^/PTEN^pos^ melanoma patients^9^. In addition, MITF has been associated with NK cell-mediated cytotoxicity in melanoma^10^. MITF promotes immune escape by inducing the expression of ADAM10, which is a direct target of MITF. ADAM10 is critical for cleavage of the NKG2D ligands MICA and MICB, which leads to reduced NK cell-mediated cytotoxicity. On the other hand, it has also been reported that MITF expression in melanoma induces NK cell cytotoxicity by inhibiting integrin beta-like protein 1 (ITGBL1) expression, which is increased in MITF-low melanoma cells and in patients with resistance to anti-PD1 therapy^11^. Furthermore, HVEM (Herpes Virus Entry Mediator; also known as TNFRSF14), a member of the TNF receptor superfamily (TNFRSF), is expressed on various types of cells: T cells, B cells, dendritic cells (DCs), NK cells and myeloid cells. HVEM interacts with BTLA (B and T lymphocyte attenuator), and activation of this axis triggers inhibitory signals in T cells to suppress antitumor immunity and is associated with poor prognosis in patients with several cancers. In addition, BTLA^+^ CD8^+^ tumor-infiltrating lymphocytes (TILs) are crucial for the efficacy of adoptive T-cell therapy in metastatic melanoma patients. In addition to showing a strong correlation with MITF expression in the skin cutaneous melanoma dataset in The Cancer Genome Atlas (TCGA), HVEM expression was recently shown to be strongly correlated with the expression of specific molecules upstream or downstream of MITF. In addition, MITF knockdown by siRNA reduced the expression of HVEM in melanoma cell lines and patient-derived melanoma cells^12,13^.

Although the relationship between MITF and PD-L1 in cancer cells remains unclear^14–16^, some evidence suggests that MITF is critical for nonresponse to ICI therapy in melanoma patients^17,18^. It has been reported that MITF is frequently expressed in the BRAF-mutant subtype of cutaneous melanoma, as determined by TCGA data analysis. Interestingly, the “MITF-low” cluster of cells in that study exhibited low expression of genes associated with immunomodulation, adhesion, migration, the extracellular matrix and proper pigmentation. Furthermore, enrichment of microRNA (miR)-100-5p and miR-125b-5p was detected in the “MITF-low” cluster of melanoma samples in TCGA, and among the evaluated tumor patients, the expression of these miRs was higher in the immunotherapy responders than in the nonresponders^15,18^. In addition, indoleamine 2,3-dioxygenase 1 (IDO1), an interferon-gamma (IFN-γ)-induced enzyme, promotes the infiltration of regulatory T cells (Tregs) and MDSCs in the tumor microenvironment (TME). Although IDO inhibitors lead to a reduction in solid tumor progression in the context of immunotherapy, they show limited therapeutic efficacy when administered in combination with anti-PD-1 antibodies. IDO1 inhibitors restore tryptophan and MITF levels, which are reduced in melanoma cells, and lead to the loss of susceptibility of these cells to the antitumor effect of T-cell-derived IFN-γ. Indeed, the expression levels of MITF and its target genes were found to be inversely correlated with the IFN-γ expression level in a TCGA melanoma cohort, and patients with tumors that responded well to immunotherapy had lower levels of MITF and the MITF target genes than patients with nonresponding tumors^19^. MiTF/TFE translocation-associated renal cell carcinoma (tRCC) is a rare subtype of non-clear cell renal cell carcinoma (nccRCC), and patients with tRCC have poorer outcomes than patients with other RCC subtypes. However, the effects of ICIs on tRCC are contradictory, and limited data are available^12,20^.

MRGPRX2 is a G protein-coupled seven transmembrane domain receptor that is expressed mainly in MCs and neurons. MRGPRX2 is associated with skin immunity, pain and adverse drug reactions. Recently, MRGPRX2 activation with substance P was shown to promote the nuclear translocation of lysyl t-RNA synthetase (LysRS) and induce MITF activation. Furthermore, several drugs known to mediate MRGPRX2-dependent degranulation, including atracurium, vancomycin and morphine, induce MITF activity. These results demonstrate that MITF and MITF-dependent targets may be therapeutic targets for pathologies associated with MRGPRX2^3^.

These findings increase our understanding of the interaction between MITF and the immune system and may provide valuable insights for the development of cancer therapies.

### MITF in immune cells

MITF is expressed in nonmelanoma cell types and influences their development and function. MITF is associated with cells of the osteoclast lineage, including osteoclast precursors, monocytes and macrophages. Stimulation of receptor activator of nuclear factor κB ligand (RANKL), a key signaling pathway protein that promotes osteoclast development, increases MITF expression and induces osteoclastogenesis^21,22^. The interactions between MITF and osteoclast-specific genes, such as tartrate-resistant acid phosphatase (TRAP) and cathepsin K (Ctsk), are essential for osteoclast differentiation and function^23,24^. MITF is also involved in MC development through the mast cell protease (MCP), granzyme B, tryptase and c-kit signaling pathways, which are required for MC activation^25–27^. In addition, the MITF inhibitor ML-329 has been shown to alter calcium influx and MC degranulation^3^. The role of MITF in maintaining the mature and quiescent state of B cells has also been demonstrated. Disruption of MITF expression induces B-cell differentiation into plasma cells and increases autoantibody production via inhibition of IRF4 expression^28^. Mutant MITF can also reduce NK cell activity by inhibiting perforin transactivation^29^. Recent evidence suggests that MITF is expressed predominantly in tumor-associated myeloid-derived suppressor cells (MDSCs) to regulate their differentiation and immunosuppressive functions^30^. Although numerous studies have investigated the mechanisms underlying MITF expression, the effects of MITF signaling pathways on immune cell development have not been fully elucidated. In this context, we further discuss the role of upstream and downstream molecules in MITF signaling pathways, especially in myeloid cells and other immune cells.

## The upstream regulators of MITF in myeloid cells

**Fig. 2:**
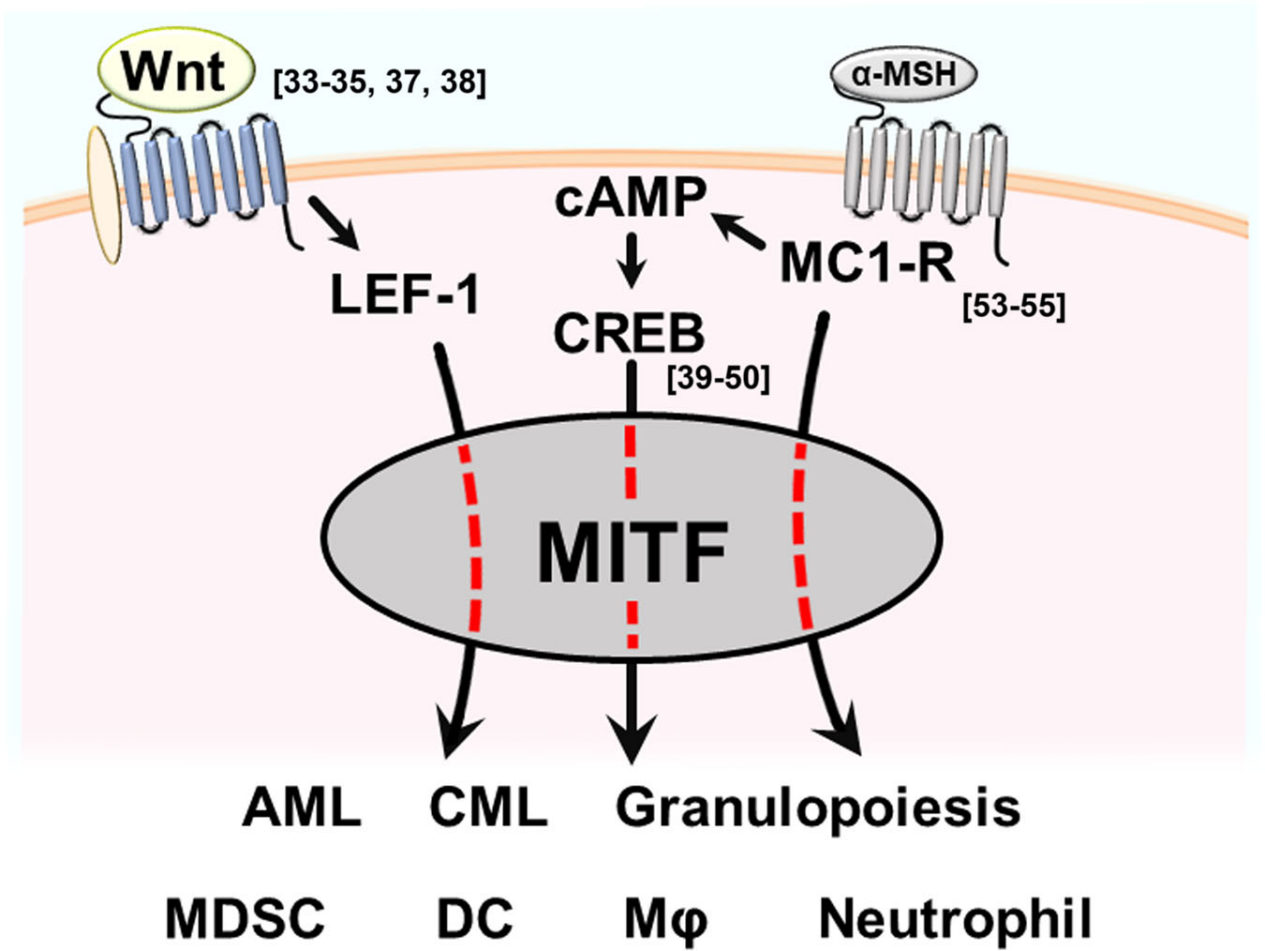
The schematic shows MITF upstream molecules and their immunological activities. MITF upstream molecules such as Wnt/LEF-1, α-MSH/MC1-R and CREB are known to have immunological functions in various immune cells and immune-related diseases. (AML acute myeloid leukemia/CML chronic myeloid leukemia).

MITF is involved in numerous cellular mechanisms through its transcriptional activity, and its function is affected by various signaling pathways and upstream molecules, including the Wnt pathway, CREB, MC1R, ATF4, BRAF, PAX3, IL-6 and TGF-β^31^. Interestingly, many of these pathways and molecules are known to have immunological functions in various immune cells and immune-related diseases (Fig. 2).

### Wnt signaling and MITF expression

The Wnt signaling pathway is one of the major pathways regulating MITF expression through the nuclear mediators, lymphoid enhancer-binding factor-1 (LEF-1)/T-cell factor (TCF) transcription factors^32^. Wnt signaling is activated in human myeloid leukemia, and the Wnt signaling mediator LEF-1 plays an important role in leukemogenesis. Furthermore, nuclear localization of β-catenin is frequently observed in human leukemia patients, and LEF-1 is critical for the nuclear localization of β-catenin in both AML cell lines and primary AML blasts. Overexpression or constitutive activation of *Lef-1* in mouse bone marrow leads to profound disruption of hematopoietic cell differentiation and to the development of acute myeloid leukemia (AML)^33,34^. In addition, LEF-1 expression has been shown to be increased in CD34^+^ bone marrow cells and peripheral blood cells from patients with blast crisis (BP) of chronic myeloid leukemia (CML)^35^. In contrast, depletion of LEF-1 was found to result in defective granulopoiesis in the context of congenital neutropenia. LEF-1 is an essential protein for neutrophil granulopoiesis because it regulates proper cell lineage commitment and granulocyte differentiation and proliferation via the actions of cyclin D1, c-myc, survivin and C/EBPα, downstream effectors of MITF^31,36,37^. It was also observed that Wnt1 induced the expression of CD36 after TCF and PPAR-γ activation, leading to the differentiation of human peripheral blood monocytes into macrophages^38^.

### Cyclic AMP response element-binding protein (CREB) is a key regulator

The activation of cyclic AMP response element-binding protein (CREB), a basic leucine zipper (bZIP) transcription factor, is induced by an elevated cAMP level, and CREB functions as a key regulator of MITF expression^32^. CREB plays various roles in immune cells, including macrophages, DCs, neutrophils, T cells and B cells, via regulation of cell proliferation, survival, differentiation and activity and is associated with many diseases^39,40^. Transgenic (TG) mice with myeloid-specific overexpression of CREB spontaneously develop skin abscesses or dermatitis, and neutrophils have been observed in mice with preputial gland abscesses. In these mice, nicotinamide adenine dinucleotide phosphate (NADPH) oxidase activity is increased in neutrophils, but neutrophil migration is not altered. In addition, overexpression of CREB in myeloid cells leads to altered cytokine levels. Overexpression of CREB reduces keratinocyte-derived cytokine (KC) and interleukin (IL)-1β levels while inducing IL-1RA, IL-10 and nuclear factor kappa B (NF-κB), which is associated with macrophage chemotaxis and function and the differentiation of progenitor cells into bone marrow-derived macrophages (BMDMs), in CREB TG mice^41^. Interestingly, CREB-1 expression is upregulated during macrophage differentiation, and CREB phosphorylation by macrophage colony-stimulating factor (M-CSF) induces the expression of PU-Ets related-1 (PE-1)/Mitogenic Ets transcriptional suppressor (METS), which contributes to growth arrest by reducing the transcription rate of cell cycle-controlling genes. Furthermore, expression of a dominant negative form of CREB-1 in bone marrow progenitors results in reduced expression of CD54 and impairs cell adhesion^42^. It has also been reported that CREB is activated in primary human macrophages infected with *Mycobacterium tuberculosis (Mtb)*, an intracellular human pathogen, and induces immune evasion and bacterial growth by reducing the fusion of Mtb-containing phagosomes and lysosomes^43^. Recently, it was reported that the neuronal guidance protein netrin-1 produced in MC38 murine colon cancer cells promotes the immunosuppressive activity of MDSCs. Netrin-1 binds to adenosine receptor 2B (A2BR), the receptor for netrin-1, on the surface of MDSCs, and this netrin-1/A2BR axis induces the suppressive effect of MDSCs on T-cell proliferation by activating the cAMP/protein kinase A (PKA)/CREB signaling pathway. In addition, MDSC infiltration is reduced in netrin-1-knockdown MC38 tumor-bearing mice^44^. Similarly, CREB-2/ATF4 has been shown to be necessary for the polarization and maturation of macrophages and MDSCs in both humans and mice. The general control nonderepressible 2 (GCN2) kinase promotes the expression of CREB-2/ATF4 and induces the acquisition of an immunosuppressive phenotype by TAMs and MDSCs in the TME. In addition, B16F10 cell-inoculated mice with myeloid-specific GCN2 knockout (B6.*Gcn2*^*fl/fl*^x*Lyz2*^*+/Cre*^ mice) exhibit decreased infiltration of TAMs and increased infiltration of CD8^+^ T cells, NK cells and NKT cells^45^. In dendritic cells, the production of the anti-inflammatory cytokine IL-10 is induced by CREB activation through a C-type lectin receptor-mediated signaling pathway^46^. Moreover, CREB affects inflammatory cytokine production in neutrophils and granzyme C expression in MCs^47,48^. Like Wnt signaling, CREB signaling affects the development of AML^49^. CREB is highly expressed in AML patients and is activated by the scaffolding adapter Gab2 via the SHP-2/Erk pathway. In addition, the selective CREB inhibitor 666-15 reduces the migration of THP-1 human AML cells by inhibiting the expression of MMP2 and MMP9, which are downstream targets of CREB^50^.

### Melanocortin 1 receptor (MC1-R) and MITF

Melanocortin 1 receptor (MC1-R) is expressed mainly in melanocytes, and the MC1R/cAMP/MITF axis plays an important role in the growth, survival and differentiation of melanocytes and melanoma cells. However, many reports have shown that MC1-R is expressed on leukocytes and antigen-presenting cells (APCs), including monocytes, macrophages, neutrophils, DCs, cytotoxic T cells, B lymphocytes and monocytic cell lines such as THP-1 and RAW264.7, and plays important roles in immune responses and inflammatory diseases. MC1-R exerts anti-inflammatory effects on all major forms of inflammation, including acute, chronic systemic, allergic and CNS inflammation, and is activated by its ligand, α-melanocyte-stimulating hormone (α-MSH), which is produced through the posttranslational processing of proopiomelanocortin (POMC)^51–53^. Atherosclerosis is a chronic inflammatory disease caused by uncontrolled cholesterol accumulation and chronic inflammation within the arterial wall, and it is associated with increased recruitment of circulating leukocytes, particularly monocytes, to inflamed arteries. Rinne et al. reported a positive correlation between MC1-R expression on macrophages and the expression of the reverse cholesterol transporters ABCA1 and ABCG1 in atherosclerotic lesions in both humans and mice. α-MSH/MC1R activation induces cholesterol efflux and reduces cholesterol uptake by BMDMs, which is a counterregulatory response to foam cell formation, and increases the levels of ABCA1 and ABCG1. In addition, treatment with the selective MC1-R agonists LD211 and MSG606 reduces lipid accumulation in BMDMs. These results suggest that targeting MC1-R on macrophages is a possible atherosclerosis therapy^54^. A single-base deletion mutation in the *MC1R* gene^(Mc1re/e)^ in mice was found to exacerbate atherosclerosis by disrupting cholesterol and bile acid metabolism and by inducing arterial recruitment of proinflammatory Ly6C^high^ monocytes^55^.

## The downstream effectors of MITF in myeloid cells

### HIF-1α as a downstream effector of MITF

**Fig. 3:**
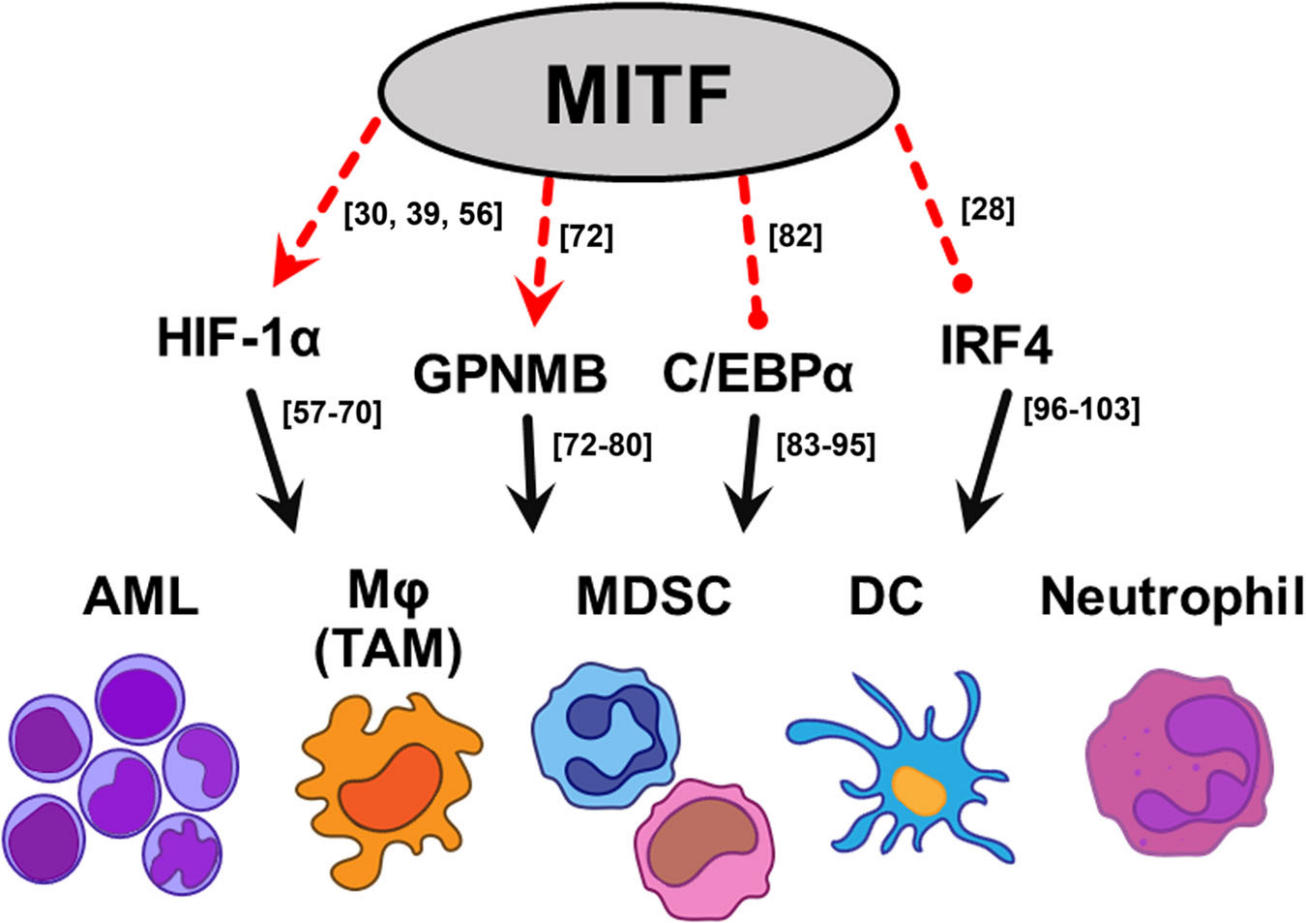
Schematic of MITF downstream targets and their role in myeloid cells. Many of the MITF downstream effectors (e.g., HIF-1α, GPNMB, C/EBPα and IRF4) are associated with the differentiation and functions of immune cells, including macrophages, MDSCs, DCs and neutrophils.

The expression of numerous target genes, including those that encode proteins involved in cell cycle arrest, proliferation, cell survival, oxidative stress, metastasis and invasion, is controlled by MITF. MITF transcriptionally regulates the expression of several downstream effectors, including AP-1, HIF and C/EBP^31^ (Fig. 3). We recently discovered that altered MITF expression in MDSCs downregulates hypoxia-inducible factor-1α (HIF-1α) expression and stimulates T-cell proliferation^30^. HIF-1α is a central transcription factor that modulates cellular responses to hypoxic conditions and plays critical roles under a variety of physiological and pathological conditions^39^. HIF-1α is a key molecule downstream of MITF and is directly influenced by MITF at the transcriptional level^56^. In addition, emerging evidence suggests that HIF-1α regulates myeloid cell differentiation and function. The HIF-1α level is increased in tumors, and HIF-1α increases the expression of arginase 1 (Arg1) and inducible nitric oxide synthase (iNOS or NOS2) in MDSCs^57^. Furthermore, HIF-1α is a direct regulator of programmed death-ligand 1 (PD-L1), which is associated with the immunosuppressive activity of MDSCs and induces MDSC-mediated decreases in T-cell function and proliferation^58^. Under hypoxic conditions, HIF-1α plays an important role in the polarization of macrophages into M2-like tumor-associated macrophages (TAMs)^59^. In addition, low oxygen levels affect the differentiation and maturation of DCs. Several studies have shown that HIF-1α induces DC-mediated inflammation and chemokine receptor type 7 (CCR7)-mediated migration of myeloid DCs (mDCs)^60–62^. In contrast, exposure to hypoxia has been shown to either inhibit or exert no effect on the proinflammatory function of DCs^63,64^. The HIF-1α level is also elevated in activated neutrophils^65^. A neutrophil extracellular trap (NET) is a web-like structure composed of chromatin and antimicrobial proteins released by activated neutrophils to trap and kill pathogens. Under hypoxic conditions, HIF-1α promotes NET formation and increases NET release^66^. AML is a complex hematological malignancy characterized by abnormal proliferation and impaired differentiation of myeloid progenitor cells. In AML, HIF-1α plays a key role in the dysregulation of myeloid cell differentiation. Altered HIF-1α expression and activity have been observed in AML cells and contribute to the disruption of myeloid cell differentiation. HIF-1α can interfere with the expression and function of key transcription factors involved in myeloid cell differentiation, such as PU.1, C/EBPα and Runx1^67,68^. Several strategies for pharmacologically targeting HIF-1α to restore normal myeloid cell differentiation in the context of AML have been reported. In patients with acute promyelocytic leukemia (APL), the combination of HIF-1α inhibitors with differentiation-inducing agents such as all-trans retinoic acid (ATRA) or arsenic trioxide (ATO) may have synergistic effects on promoting myeloid cell differentiation and improving treatment outcomes^69,70^.

### GPNMB (DC-HIL) and MITF

Glycoprotein NMB (GPNMB, a DC-associated transmembrane protein; also known as DC-HIL/osteoactivin) is an endogenous type 1 transmembrane glycoprotein that was initially shown to be highly expressed in melanoma cell lines with low metastatic capacity^71^. MITF has also been identified as a key regulator of GPNMB expression in DCs. Treatment of monocyte-derived dendritic cells (moDCs) with small molecule inhibitors of MITF reduces GPNMB expression at both the mRNA and protein levels, suggesting that GPNMB expression is promoted by MITF activation^72^. Interestingly, GPNMB expression is greatly increased by IL-10 and BCR-ABL tyrosine kinase inhibitors (imatinib, nilotinib and dasatinib), which are used to treat CML^73^. In addition, IL-10 induces the activation of MITF, which inhibits the activity of the transcription factor NF-κB, resulting in its inactivation. This inhibition of NF-κB activation reduces the production of costimulatory molecules, major histocompatibility complex (MHC) molecules and IL-12^73,74^. Therefore, anti-GPNMB treatment induces the expression of CD80, CD86 and C/EBP in DCs and increases the production of the proinflammatory cytokines IL-1β and TNF-α. In addition, GPNMB inhibition may prevent T cells from binding to syndecan-4 (SD-4), resulting in increased T-cell activity^75^. Understanding the effects of GPNMB on surface marker expression and cytokine production may provide insights into its role in regulating DC-mediated immune activation and tolerance. The migration and homing of DCs to lymphoid organs are essential for the inhibition of immune responses. Studies suggest that GPNMB may affect the ability of DCs to migrate. The expression of the chemokine CC motif ligand (CCL19) and CCR7, which are important for DC migration into tissues, is regulated by GPNMB expression on DCs. In addition, GPNMB affects the expression of adhesion molecules and extracellular matrix components involved in DC migration and interactions with lymphoid tissues^75^. Thus, understanding the effects of GPNMB on DC migration and homing is expected to provide insights into its potential role in altering immune cell dynamics and immune surveillance.

GPNMB is expressed on macrophages and is more abundant on M2 macrophages than on M1 macrophages. GPNMB promotes M2 polarization, suggesting that it may facilitate the resolution of inflammation^76^. Although many studies have suggested that GPNMB may function as an anti-inflammatory molecule by promoting the resolution of inflammation, there is evidence suggesting that it may promote inflammation. Hence, the function of GPNMB in inflammation remains unclear. Several studies have shown that the GPNMB level is elevated in tissues under inflammatory conditions, such as those caused by LPS treatment, and in pathological contexts, such as neurodegenerative diseases and cancer^71^. There is also evidence that a novel paracrine axis comprising GPNMB and IL-33 is activated by interactions between macrophages and tumor cells, ultimately promoting tumor cell survival, cancer stem cell expansion and metastasis development^77^. In addition, GPNMB is regulated by epigenetic mechanisms and has been identified as a target gene of enhancer of zeste homolog 2 (EZH2). Since EZH2-mediated H3K27me3 modification is associated with gene silencing, when EZH2 is suppressed in macrophages, the GPNMB level is elevated, resulting in macrophage polarization toward the M2 type. However, EZH2 knockout reduces neutrophil recruitment and impairs bacterial clearance^76,78^. Intriguingly, GPNMB is expressed on CD14^+^ M-MDSCs and binds SD-4, which is expressed on T cells, to effectively inhibit T-cell activation^79^. Since increasing T-cell activity by blocking MDSC function is an attractive approach for cancer immunotherapy, after GPNMB was identified as a T-cell inhibitory receptor that mediates the suppressive function of MDSCs, the potential of anti-GPNMB mAbs as MDSC-targeted cancer therapeutics has been evaluated in several studies^80^.

### C/EBPα activity modulated by MITF

CCAAT/enhancer-binding protein α (C/EBPα) is a transcription factor belonging to the leucine zipper (LZ) family. C/EBPα is essential for myelopoiesis, and its function has been shown to be inhibited by MITF. It is encoded by the *CEBPA* gene and is expressed as the C/EBPαp42, C/EBPαp30 and C/EBPαLZ isoforms^81,82^. C/EBPα functions as a transcriptional activator or repressor depending on the target gene and the cell in which it is expressed. C/EBPα is critical for the maturation of myeloid progenitor cells into granulocytes and monocytes during hematopoiesis, but in the context of AML, altered C/EBPα expression disrupts this process, leading to the accumulation of immature myeloid cells and blockade of cell differentiation. Previous studies have revealed *CEBPA* open reading frame (ORF) mutations in 10-15% of AML patients^83^. The expression of C/EBPα is decreased in AML patients, possibly as a result of various mechanisms, such as epigenetic alterations, transcriptional dysregulation or deregulation of signaling pathways. In contrast, increased expression of C/EBPα has been observed in some AML patients due to chromosomal rearrangements or alterations in regulatory elements^84,85^. Therefore, pharmacological approaches, gene therapy and genetic manipulation may be used to restore C/EBPα function and promote myeloid differentiation. Numerous studies in which C/EBPα is targeted with a combination of therapeutic agents have reported promising results for the treatment of AML. Synergistic effects have been observed when agents such as HDACis or DNMTis that restore the expression of C/EBPα signature genes are combined with other targeted agents, such as tyrosine kinase inhibitors (TKIs) or inhibitors of signaling pathways associated with AML^86–88^. Increasing C/EBPα expression via treatment with small molecules in combination with anticancer drugs can be used to treat AML. Recently, MTL-CEBPA, a small activating RNA drug that increases the C/EBPα level, was shown to promote myeloid cell differentiation into neutrophils and contribute to the inhibition of proinflammatory cytokine production^89^.

Upregulation of C/EBPα expression exerts anticancer effects by inhibiting the activity of suppressive myeloid cells, such as monocytic MDSCs (M-MDSCs) and TAMs^90^. C/EBPα cooperates with other transcription factors, such as PU.1 and IRF8, to promote the differentiation of progenitor cells into the macrophage lineage^91^. C/EBPα influences macrophage polarization by regulating the production of pro- and anti-inflammatory cytokines. Knockdown of C/EBPα in macrophages induces their polarization into M1 macrophages, which are characterized by the production of proinflammatory cytokines, such as TNF-α and IL-1β^92^. C/EBPα expression is also downregulated in the early stages of MDSC development, when these cells acquire immunosuppressive properties. C/EBPα and C/EBPβ form heterodimers and bind to the promoter regions of genes such as Arg1 and iNOS, which are associated with the immunosuppressive activity of MDSCs^93^. Strategies to control C/EBPα activity in MDSCs may increase antitumor immune responses in cancer. In addition, combining C/EBPα modulation with other therapeutic approaches, such as anti-PD-1 therapy, may enhance the efficacy of these treatments by reducing the immunosuppressive effects mediated through the tumor microenvironment^94,95^.

### IRF4 and MITF

Interferon regulatory factor 4 (IRF4) has been reported to regulate the differentiation and function of MDSCs. Inhibition of IRF4 expression in MDSCs can lead to increased expression of immunosuppressive molecules, such as Arg1, iNOS, IL-10 and TGF-β. In addition, MDSCs lacking IRF4 suppress T-cell proliferation^96^. Interestingly, IRF4 is a known transcriptional target that is repressed by MITF^28^. IRF4 can affect the expression of cell surface molecules, including PD-L1, on MDSCs. IRF4-mediated PD-L1 downregulation prevents T-cell exhaustion and attenuates immune tolerance through the PD-1/PD-L1 signaling pathway. Furthermore, IRF4 can affect the levels of inflammatory factors, including IL-1α and IL-10, thereby modulating the immunosuppressive microenvironment^96,97^. Targeting these proteins that cooperate through their interactions may provide therapeutic opportunities to modify MDSC function and restore immune homeostasis in patients with diseases characterized by MDSC-mediated immune dysregulation.

IRF4 is involved in the polarization of macrophages into different types with distinct functions. IRF4 expression is often downregulated in M1 macrophages, promoting the expression of M1-specific genes involved in proinflammatory responses. IRF4 can be mechanistically inhibited by IRF5, which promotes M1 polarization. IRF4 inhibition is also associated with the upregulation of TNF-α, IL-1β, iNOS and CCL2, which promote macrophage polarization toward the M1 phenotype. IRF4 promotes the differentiation of monocytes into M2 macrophages by regulating the expression of M2-related genes and production of cytokines, including Arg1, Fizz1, Ym1, IL-10 and IL-4^98,99^. In addition, IRF4 influences the phenotypic and functional changes that occur during DC maturation. In DCs, IRF4 inhibits the production of IFN-γ and IL-12, which control Th1 immune responses^100,101^. IRF4 expression is also required for the maturation of conventional type 2 DCs (cDC2s) and affects DC migration involved in T-cell priming^102^. IRF4 also affects the development and survival of intestinal CD103^+^CD11b^+^ DCs, which are involved in antimicrobial defense^103^. The novel functions of IRF4 in DCs can be leveraged for cancer immunotherapy, providing new therapeutic interventions. Targeting IRF4 is expected to enhance DC-mediated antitumor immune responses, stimulate the priming of tumor-specific T cells and inhibit immunosuppressive mechanisms mediated through the TME.

## MITF cofactors in myeloid cells

The differentiation and function of myeloid cells are controlled by a complex network of transcription factors. PU.1 is an ETS family transcription factor that plays multiple roles in hematopoiesis. It is a direct regulator of myeloid cell, DC and B-cell functional programs and a well-characterized molecule in terminal erythroid cell differentiation. MITF and PU.1 have emerged as key players in myeloid cell development. They play an essential role in monocyte differentiation into macrophages and function cooperatively to increase the expression of target genes, such as Ctsk, acid phosphatase 5 (Acp5), c-Fos and nuclear factor of activated T cells (NFATc1), during osteoclast differentiation^104^. They also function in concert with Eomesodermin (EOMES) to regulate the activity of transcription factors important for osteoclast differentiation. This action is accomplished by the binding of MITF and PU.1 to the promoter region of the gene, where transcription factors regulate the expression of the gene^105^. PU.1 is essential for cDC maturation and function and inhibits plasmacytoid DC (pDC) differentiation by directly activating the expression of DC-SCRIPT (Zfp366)^106^. In addition, PU.1 plays a critical role in DC migration to secondary lymphoid organs by inducing the expression of CCR7. In fact, it has been reported that PU.1 regulates CCR7 expression in both mouse and human DCs by binding to the Ets motif^107^.

MITF also binds to NFATc1, which regulates myeloid cell differentiation and immune responses. NFATc1 has been previously shown to be expressed in T cells, MCs and osteoclasts and to regulate the action of several transcription factors. In particular, NFATc1 is important for IL-2 and IL-4 production in T cells. Interestingly, NFATc1 is also expressed in MDSCs and has been shown to enhance the immunosuppressive function of MDSCs via its effect on IDO expression induced by treatment with cyclosporine (CsA), a calcineurin inhibitor. The NFAT inhibitory peptide VIVIT has also been shown to inhibit T-cell differentiation by promoting the suppressive activity and recruitment of MDSCs^108^. In addition, NFATc1 has been shown to be expressed in mouse MCs and to be involved in MC survival and apoptosis by binding to the IL-13 and TNF-α promoters, activating the expression of the respective genes^109^.

## Conclusion

Previously, MITF was shown to be a key gene regulator involved in melanocyte and melanoma cell functions, affecting skin color regulation, melanoma growth, immunity and melanoma progression. However, recent studies suggest that the role of MITF may extend beyond melanoma cells and melanocytes to other cell types. MITF regulates the differentiation and function of several immune cells in addition to osteoclasts, including MDSCs, and as a transcription factor, it is involved in immune responses via its regulation of multiple signaling pathways through interactions with various partners. Although various studies have reported MITF expression in different cell types, the mechanism underlying its function, as well as its precise role in immune cells, are still unclear. In this review, we focused on identifying representative upstream and downstream signaling mediators of MITF that modulate immune responses mediated by various hematopoietic cells. In particular, MITF has been shown to play an important role in regulating the differentiation and immunosuppressive function of MDSCs in the TME. MITF expression induced by soluble factors in the TME increases the immunosuppressive activity of MDSCs, especially M-MDSCs, promotes MDSC differentiation and inhibits CD8^+^ T-cell proliferation. These effects may be associated with poor survival in breast and lung cancer patients, and modulation of MITF expression may be a breakthrough strategy to restore T-cell activity and increase the efficacy of anticancer therapy^30^. Unfortunately, although the expression and function of MITF in various diseases and immune cells have been actively studied, research on the use of MITF as a therapeutic target was lacking until recently, and inhibitors targeting MITF have not been developed. Thus, a better understanding of the interactions between MITF and the immune system may help to improve the clinical benefits and the long-lasting effects on immune regulation or antitumor immune responses.
